# Detecting association of rare and common variants by testing an optimally weighted combination of variants with longitudinal data

**DOI:** 10.1186/1753-6561-8-S1-S91

**Published:** 2014-06-17

**Authors:** Shuaicheng Wang, Shurong Fang, Qiuying Sha, Shuanglin Zhang

**Affiliations:** 1 Department of Mathematical Sciences, Michigan Technological University, 1400 Townsend Drive, Houghton, MI 49931, USA

## Abstract

Increasing evidence shows that complex diseases are caused by both common and rare variants. Recently, several statistical methods for detecting associations of rare variants have been developed, including the test for testing the effect of an optimally weighted combination of variants (TOW) developed by our group in 2012. These methodologies consider phenotype measurement at only one time point. Because many sequence data have been developed on population cohorts that contain phenotype measurements at multiple time points, such as the data set provided in the Genetic Analysis Workshop 18 (GAW18), we extend TOW from phenotype measurement at one time point to phenotype measurements at multiple time points. We then apply the newly proposed method to the GAW18 data set and compare the power of the new method with TOW using only one phenotype measurement. The application results show that the newly proposed method jointly modeling phenotype measurements at all time points has increased power over TOW.

## Background

There is increasing interest in detecting associations between rare variants and complex traits. Although statistical methods to detect common variant associations have been well developed, these variant-by-variant methods may not be optimal for detecting associations of rare variants as a result of allelic heterogeneity as well as the extreme rarity of individual variants [1]. Recently, several statistical methods for detecting associations of rare variants have been developed, including the cohort allelic sums test [2], the combined multivariate and collapsing method [1], the weighted sum statistic [3], and the variable minor allele frequency threshold method [4], among others. These methods are essentially testing the effect of a weighted combination of variants. Thus, choosing appropriate weights is critical to the performance of these methods. In Sha et al [5], we proposed a novel test for testing the effect of an optimally weighted combination of variants (TOW). The optimal weights are analytically derived. Based on the optimal weights, TOW tests the effect of a weighted combination of variants. Simulation studies showed that TOW performed better than the existing methods across a wide range of scenarios. Aforementioned methods are for phenotypes at a single time point and cannot be applied to longitudinal phenotypes directly.

Meanwhile, quite a few statistical methods on the analysis of longitudinal data in the context of genetic mapping and association studies have been developed for common variants [6-10]. A typical method is functional mapping, which uses mathematical models to connect the actions of genes and the development of a trait. Several mathematical functions have been established to describe the development of a phenotype, including parametric functions [6], semiparametric functions [8], and nonparametric functions [9]. From a statistical standpoint, any modeling using longitudinal phenotypes is more informative than that using phenotypes at a single time point and thus can increase power to test association [7,10]. Functional mapping capitalizes on the full information provided by growth and development of phenotypes over time, increasing the power of gene identification. However, no statistical methods on the analysis of longitudinal data are available for rare variants.

To analyze the sequencing data with phenotype measurements at multiple time points provided by Genetic Analysis Workshop 18 (GAW18) [11], in this article, we propose a novel method to test rare-variant association with longitudinal phenotypes by extending our previously proposed method, TOW. Applying the proposed method to the GAW18 data set, we compare the power of the proposed method with TOW using only one phenotype measurement.

## Methods

Consider a random sample of *n *individuals. Each individual has been genotyped at *M *variants in a genomic region (a gene or a pathway). Denote $\left(x_{i1},...,x_{iM}\right)$ as the genotypic score of the *i*th individual, where $x_{im}\in \left\{0,1,2\right\}$ is the number of minor alleles. Let $x_{i}=\overset{M}{\underset{m=1}{\sum }}w_{m}x_{im}$ denote the weighted combination of genotypic scores at the M variants, where $w_{1},\ldots ,w_{M}$ are unknown constants and their values are determined later using some optimal criteria. For longitudinal data, we assume that phenotypes and covariates are collected at *K *time points. Let $y_{ij}$ and $z_{ij}={\left(z_{ij1},\ldots ,z_{ijP}\right)}^{T}$ denote the trait values and the covariates of the *i*th individual at the *j*th time point. For longitudinal data, we propose a mixed linear model to model the relationship between phenotype, covariates, and genotypic scores:

$y_{ij}=Z_{ij}^{T}\alpha +\beta x_{i}+v_{ij}+e_{ij}$,

where $Z_{ij}={\left(1,z_{ij}^{T}\right)}^{T}$ and $\alpha ={\left(\alpha_{0},\ldots ,\alpha_{P}\right)}^{T}$; $v_{ij}$ is a random variable representing the environmental contribution to the phenotype and $e_{ij}$ is a random error term.

The matrix form of this mixed linear model can be written as

(1) y=Zα+xβ+v+e,

where $y={\left(y_{11},...,y_{1K},\cdots \;,y_{n1},...,y_{nK}\right)}^{T}$, $Z={\left(Z_{11},...,Z_{1K},\cdots \;,Z_{n1},...,Z_{nK}\right)}^{T}$, $x={\left(x_{1},...,x_{1},\cdots \;,x_{n},...,x_{n}\right)}^{T}$, *v *is the vector form of $v_{ij}$, and *e *is the vector form of $e_{ij}.$ We assume that *e *follows normal distribution $N\left(0,\sigma_{e}^{2}I\right)$ and *v *also follows normal distribution $N\left(0,\sigma_{v}^{2}D\right)$, where $D=diag\left(D_{0},\ldots ,D_{0}\right)$ and $D_{0}$ depends on the level of correlation of phenotypes between time points. The total variance of *y *is $\text{Σ}=\sigma_{v}^{2}D+\sigma_{e}^{2}I$. Following Furlotte et al [10], we use sample correlation coefficients of phenotypes between time points to estimate $D_{0}$. For variance components $\sigma_{v}^{2}$ and $\sigma_{e}^{2},$ we use maximum likelihood estimates (MLEs) under null hypothesis $H_{0}:\beta =0$ as estimates of $\sigma_{v}^{2}$ and $\sigma_{e}^{2}$ and impute the estimated values of $\sigma_{v}^{2}$ and $\sigma_{e}^{2}$ into model (1). Let ${\hat{\sigma }}_{v}^{2}$ and ${\hat{\sigma }}_{e}^{2}$ denote the MLEs under null hypothesis of $\sigma_{v}^{2}$ and $\sigma_{e}^{2}$, and let $\hat{\text{Σ}}={\hat{\sigma }}_{v}^{2}D+{\hat{\sigma }}_{e}^{2}I$. After imputing the estimated values of $\sigma_{v}^{2}$ and $\sigma_{e}^{2}$, model (1) becomes

(2) y=Zα+xβ+ϵ,

where *ϵ *follows $N\left(0,\hat{\text{Σ}}\right)$.

Let $y_{T}={\hat{\text{Σ}}}^{-1/2}y$, $x_{T}={\hat{\text{Σ}}}^{-1/2}x$, and $Z_{T}={\hat{\text{Σ}}}^{-1/2}Z$. Then model (2) is equivalent to

(3) $$y_{T}=Z_{T}\alpha +x_{T}\beta +\epsilon_{T},$$

where $\epsilon_{T}$ follows N(0,I). The score test statistic under model (3) to test null hypothesis $H_{0}:\beta =0$ is given by

$T_{score}=\frac{{\left(y^{*T}x^{*}\right)}^{2}}{{\hat{\sigma }}^{2}x^{*T}x^{*}}$,

where $y^{*}$ and $x^{*}$ are the residuals under models $y_{T}=Z_{T}\alpha +\epsilon_{T}$ and $x_{T}=Z_{T}\alpha +\epsilon_{T}$, respectively, and ${\hat{\sigma }}^{2}=\frac{1}{nK}y^{*T}y^{*}$. Let $X_{m}={\left(x_{1m},...,x_{1m},\cdots \;,x_{nm},...,x_{nm}\right)}^{T}$, $X_{mT}={\hat{\text{Σ}}}^{-1/2}X_{m}$, $X_{m}^{*}$ is the residuals under the model $X_{mT}=Z_{T}\alpha +\epsilon_{T}$, and $X^{*}=\left(X_{1}^{*},\ldots ,X_{M}^{*}\right)$. Then

$T_{score}=\frac{w^{T}X^{*T}y^{*}y^{*T}X^{*}w}{{\hat{\sigma }}^{2}w^{T}Aw}$,

where $A=X^{*T}X^{*}$.

One potential problem with the score test $T_{score}$ is that for genotype data of rare variants, it will be problematic to use *A *to estimate the covariance matrix because of sparse data. Following Pan [12] and Sha et al [5], we replace *A *by $A_{0}=diag\left(A\right)$. Then, the score test statistic is equivalent to

$T_{0}\left(w\right)=\frac{w^{T}X^{*T}y^{*}y^{*T}X^{*}w}{w^{T}A_{0}w}$.

As a function of *w*, $T_{0}\left(w\right)$ reaches its maximum when $w=w^{o}=A_{0}^{-1}X^{*T}y^{*}$ and the maximum value of $T_{0}\left(w\right)$ is $y^{*T}X^{*}A_{0}^{-1}X^{*T}y^{*}$. Based on longitudinal data, we define the statistic to test the effect of the optimally weighted combination (L-TOW) of variants, $\sum_{m=1}^{M}w_{m}^{o}x_{im}^{}$, as

$T_{L-TOW}=y^{*T}X^{*}A_{0}^{-1}X^{*T}y^{*}=\overset{M}{\underset{m=1}{\sum }}\frac{{\left(y^{*T}X_{m}^{*}\right)}^{2}}{X_{m}^{*T}X_{m}^{*}}$.

We use a permutation test to evaluate the *p-*value of $T_{L-TOW}$. In each permutation, we randomly shuffle the elements of $y^{*}$.

## Results

We chose 157 genetically unrelated individuals from the file UNREL.txt. These individuals were extracted from 20 pedigrees in GAW18. We extracted genotypes for those individuals from files named chrN-dose.csv.gz. These files provided the estimated number of minor alleles carried for each variant. We used 200 replicates of simulated phenotype data in files PHEN.#.csv, where # is replicate number 1 to 200. Sex, age, medication use, and tobacco smoking were considered as covariates in this study. The phenotype data have been simulated at three time points with no missing data. There are 15 individuals without phenotype values in the simulated phenotype data, so the actual number of individuals used in this study is 142. To get reasonable powers for the power comparison, we merged 2 replicates to form a new replicate, so the total number of replicates for power comparison in this study was 100. We know the answers of the simulated data set in this study.

**Figure 1:**
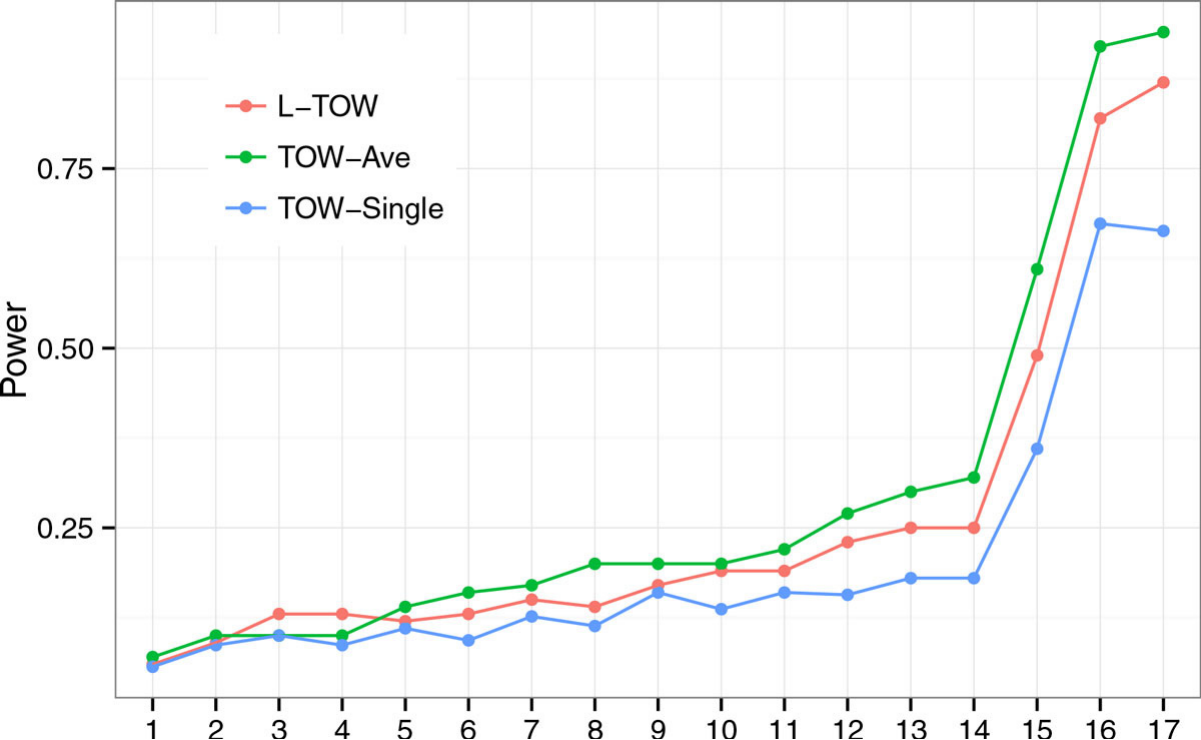
**Power comparisons of the three tests using diastolic blood pressure as a phenotype measurement**. Power of TOW-Single is the average power of TOW-1, TOW-2, and TOW-3. Numbers 1 to 17 on the x-axis refer to genes *ZNF443, ABTB1, FLNB, SLC35E2, TNN, CGN, ZFP37, LRP8, RAI1, ZNF544, LEPR, MTRR, NRF1, REPIN1, PTTG1IP, FLT3*, and *MAP4*, respectively. *TOW*, statistic to test the effect of the optimally weighted combination.

There are 2 related phenotypes, systolic blood pressure (SBP) and diastolic blood pressure (DBP) at three time points. Based on the 2 related phenotypes, we consider 4 phenotype measurements: SBP, DBP, the first principal component of SBP and DBP, and the summation of SBP and DBP. For each phenotype measurement, we consider five tests: (a) L-TOW, which uses phenotype measurements at three time points; (b) TOW-1, TOW based on phenotype measurement at the first time point; (c) TOW-2, TOW based on phenotype measurement at the second time point; (d) TOW-3, TOW based on phenotype measurement at the third time point; and (e) TOW-Ave, TOW based on the average phenotype measurements over three time points. Based on each of the 4 phenotype measurements, we compare the power of L-TOW, TOW-Ave, and TOW-Single (average power of TOW-1, TOW-2, and TOW-3) to detect association between each of the top 17 genes that influence only DBP, only SBP, or both DBP and SBP. The power comparisons based on phenotype measurement DBP are given in Figure 1. This figure shows that in 15 of 17 genes, L-TOW is the most powerful test, TOW-Ave is the second most powerful test, and TOW-Single is the least powerful one. Power comparisons based on other three phenotype measurements show similar patterns. (Results are not showed.)

**Figure 2:**
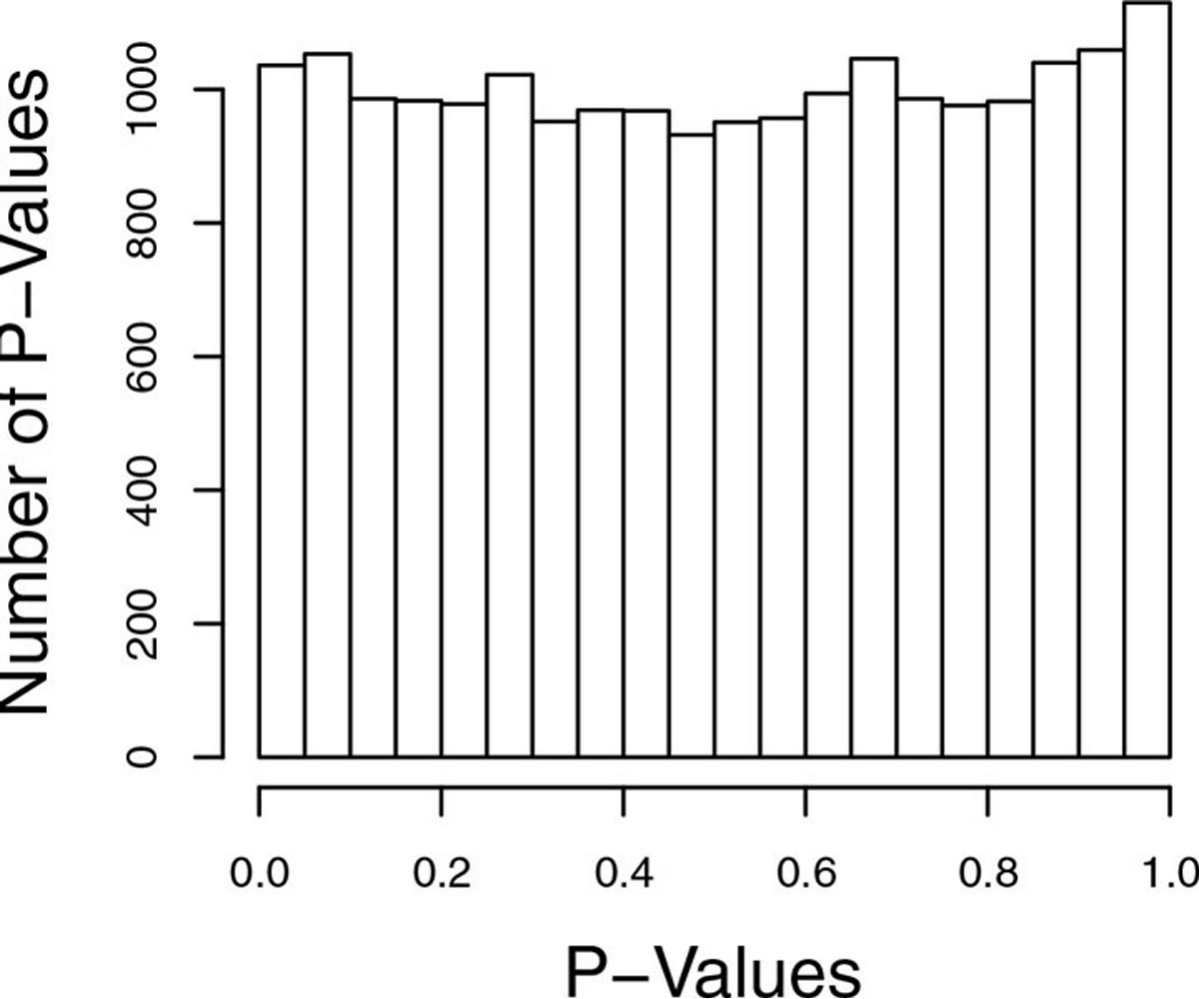
**Histogram of *p-*values**. Two hundred blocks (100 variants in each block) that are far from causal variants in chromosome 21 are chosen. In each block, the statistic to test the effect of the optimally weighted combination (L-TOW) is applied to each of the 100 replicates to test association between genotypes and the trait systolic blood pressure. One *p-*value is obtained for each replicate and each block.

We also evaluated type I error rates of the proposed test, L-TOW. To evaluate the type I error we chose 200 blocks (100 variants in each block) from chromosome 21 that are far from causal variants. In each block, we applied L-TOW to each of the 100 replicates to test association between genotypes and the trait SBP. We obtained one *p-*value for each replicate and each block. The histogram of the 20,000 *p-*values is given in Figure 2. This figure shows that the distribution of *p-*values is very close to the uniform distribution, which indicates that L-TOW has correct type I error.

## Discussion

We have developed TOW to detect association of rare and common variants [5]. Because the GAW18 data set provided phenotype measurements at multiple time points, similar to most of the existing methods for rare-variant association studies, TOW can only be applied to this data set by either using phenotype measurement at a single time point or using the average phenotype measurements over all time points. It is likely that a method jointly modeling phenotype measurements at all time points may increase power. This motivated us to extend our previously developed method, TOW, from phenotype measurement at one time point to phenotype measurements at multiple time points. By applying our newly developed method L-TOW to the GAW18 simulated data set, we showed that L-TOW has increased power over TOW by using either phenotype measurement at one time point or average phenotype measurements over multiple time points.

Although we describe our method using unrelated individuals, it is not difficult to extend the method to family-based data. For family data, denote $\left(x_{ij1},...,x_{ijM}\right)$ as genotypic score of the *j*th member in the *i*th family and $x_{ij}=\overset{M}{\underset{m=1}{\sum }}w_{m}x_{ijm}$. Let $y_{ijk}$ and $z_{ijk}={\left(z_{ijk1},\ldots ,z_{ijkP}\right)}^{T}$ denote the trait values and the covariates of the *j*th member in the *i*th family at the *k*th time point. For family data, we can use the following mixed linear model

$y_{ijk}=Z_{ijk}^{T}\alpha +\beta x_{ij}+u_{ij}+v_{ijk}+e_{ijk}$,

where $u_{ij}$ is a random variable modeling the correlation between family members, $v_{ijk}$ is a random variable modeling the correlation of phenotype measurements between time points, and $e_{ijk}$ is a random error term. Based on this model, using a similar argument to that in the Methods section, we can test association between the phenotype and the genomic region.

Comparing our method with functional mapping, whereas our proposed method uses age as a covariate and uses a single parameter *β *as the average effect over time of genotypes after adjusting for age effects, functional mapping uses mathematical models to connect gene actions and growth or development of a trait. Our proposed method has fewer parameters than the functional mapping method and uses less information. Our proposed method can easily incorporate the combination of rare variants. Incorporating the combination of rare variants to functional mapping requires further investigation.

## Conclusions

We propose a novel method to test rare-variant association with longitudinal phenotypes by extending TOW, our previously proposed method. Application to the GAW18 data set shows that the newly proposed method jointly modeling phenotype measurements at all time points has increased power over TOW, which uses only one phenotype measurement.

## Competing interests

The authors declare that they have no competing interests.

## Authors' contributions

SZ designed the overall study, SW and SF conducted statistical analyses, and QS and SZ drafted the manuscript. All authors read and approved the final manuscript.
